# Sex differences in social cognition among individuals with schizophrenia and in healthy control participants: a secondary analysis of published data

**DOI:** 10.1007/s00737-024-01422-8

**Published:** 2024-01-20

**Authors:** Anja Vaskinn, Torill Ueland, Ingrid Melle, Kjetil Sundet

**Affiliations:** 1 Centre for Research and Education in Forensic Psychiatry, Oslo University Hospital, PO Box 4956, Nydalen, 0424 Oslo Norway; 2 Institute of Clinical Medicine, Norwegian Centre for Mental Disorders Research, University of Oslo, Oslo, Norway; 3 Department of Psychology, University of Oslo, Oslo, Norway; 4 Psychosis Research Section, Norwegian Centre for Mental Disorders Research, Oslo University Hospital, Oslo, Norway

**Keywords:** Theory of mind, Emotion processing, Affect perception, Social perception, Women

## Abstract

**Purpose:**

Sex differences are present among individuals experiencing schizophrenia. Whether these differences extend to social cognition is unclear. In this study, we investigated sex differences in emotion perception, social perception and theory of mind (ToM).

**Methods:**

We examined sex differences between males and females with schizophrenia on five social cognitive tests. Healthy male and female control participants were included to examine if any sex difference was illness-specific. Emotion perception was measured with Pictures of Facial Affect (PFA) and Emotion in Biological Motion (EmoBio); social perception with the Relationships Across Domains Test (RAD); and ToM with the Movie for the Assessment of Social Cognition (MASC) and Hinting Task.

**Results:**

Two-way analyses of variance revealed overall group differences for all tests, with healthy controls outperforming individuals with schizophrenia. Significant sex effects were present for PFA and Hinting Task. There were no significant interaction effects. Within-group independent samples *t*-tests yielded one significant sex difference, i.e., among healthy controls for PFA.

**Conclusions:**

Females had better facial emotion perception than males. This sex difference was statistically significant among healthy controls and medium-large among individuals experiencing schizophrenia. There were no significant sex differences for other social cognitive domains. The study did not find evidence for a general female advantage in social cognition.

## Introduction

Social cognition has come to hold a central position in the research field’s efforts to understand schizophrenia and related psychotic disorders, both in terms of characterizing individuals experiencing schizophrenia as well as how to treat the disorder. Social cognition refers to mental operations that underlie social interactions and include the ability to perceive and interpret the intentions, dispositions, and behaviors of others (Green et al. 2008). There are different social cognitive domains, such as emotional processing, theory of mind (ToM), social perception, and attributional style. Emotional processing refers to perceiving and using emotional information and includes recognizing and labeling emotions in faces and bodies. ToM is the ability to infer the mental state of others, such as their thoughts, intentions, and emotions. Social perception has been defined as the ability to decode and interpret social cues, and attributional style refers to how we interpret and explain social events (Pinkham 2014).

Although social and nonsocial cognitive impairments form part of the clinical picture of schizophrenia and are mentioned in the latest editions of the diagnostic manuals, there is substantial cognitive heterogeneity. Several studies have identified subgroups with different levels of social cognitive performance. This usually includes one group with pronounced impairments, and one (Vaskinn et al. 2022) or two (Etchepare et al. 2019; Rocca et al. 2016) groups with milder reductions, sometimes even one group with largely intact social cognition (Hajduk et al. 2018). The sources of this heterogeneity are not fully understood. There are likely to be biological influences, as illustrated by one study that identified a genetic association with social cognitive performance (Warrier et al. 2018). In addition, factors external to the illness probably contribute to different levels of social cognitive performance. Nonsocial cognition has been linked to environmental stressors, such as childhood maltreatment, both in non-clinical populations and in psychotic disorders (Vargas et al. 2019). Preliminary evidence for psychotic disorders suggests that this may also be the case for social cognition (Rokita et al. 2018; Fares-Otero et al. 2023).

From studies conducted in the general population, we know that demographic variables are another external factor that affect social cognitive performance. One study with more than 40,000 participants found that both social class and culture explained variance on test results, albeit with differences between social cognitive tests (Dodell-Feder et al. 2020). Race also matters, in that emotion recognition is better for own-race than other-race faces (Elfenbein & Ambady 2002). Moreover, social cognitive performance differs between age groups, with a decline in performance appearing with advancing age (Olderbak et al. 2019; Dorris et al. 2022). Finally, there may be social cognitive differences based on a person’s sex. Among nonsocial cognitive functions, there is a male advantage for mental rotation (Zell et al. 2015) and a female advantage for verbal fluency and verbal episodic memory (Hirnstein et al. 2023). For social cognition, sex differences are smaller and slightly in favor of women. For emotion recognition, a small advantage in favor of females has been reported in both meta-analytic (Thompson & Voyer 2014) and large-scale community (Olderbak et al. 2019) studies. A study of the Reading the Mind in the Eyes Test (RMET), which may assess ToM or emotion perception (Oakley et al. 2016), found a female-advantage, across 57 countries and across age groups (Greenberg et al. 2023). An RMET meta-analysis found the same (Kirkland et al. 2013). Such findings from the general population suggest that sex may be among the non-illness factors affecting social cognition, contributing to the social cognitive heterogeneity seen in schizophrenia. Given that men and women with schizophrenia differ for several features of the disorder (Riecher-Rössler et al. 2018), perhaps even necessitating sex-specific treatment (Fernando et al. 2020), it is important to establish whether there are sex differences in social cognition.

Studies examining sex differences in social cognition among individuals experiencing schizophrenia have not provided consistent results. Although some find slightly better performance among women (Bozikas et al. 2006; Vaskinn et al. 2007; Abu-Akel & Bo 2013; Erol et al. 2013), many studies report no significant sex differences, whether for emotion recognition (Navarra-Ventura et al. 2021), ToM (Navarra-Ventura et al. 2021; Kubota et al. 2022) or when applying the RMET (Ayesa-Arriola et al. 2014). Similarly, several studies including a battery of social cognitive tests failed to identify any sex differences. This was the case in a recent first-episode psychosis study of emotion perception, ToM, and attributional style (Verdaguer-Rodríguez et al. 2021). Further, the Social Cognition and Functioning in Schizophrenia (SCAF) study identified sex differences on only one of five social cognitive measures (Ferrer-Quintero et al. 2021), and the Social Cognition Psychometric Evaluation (SCOPE) study found no effect of sex on social cognitive performance (Pinkham et al. 2017). In sum, sex differences in social cognition among persons living with schizophrenia appear to be modest.

We have published several studies on social cognition using different tests, without a specific focus on sex differences. Here, we present secondary analyses of sex differences in social cognition using data sets from previous publications (Vaskinn et al. 2016, 2017, 2018; Frøyhaug et al. 2019). In our initial validation study of one of our ToM measures, which used samples (Fretland et al. 2015) that subsequently have been increased (Vaskinn et al. 2018), we reported no sex differences. Based on the empirical literature reviewed above and our early ToM study, we expect to find few sex differences.

## Materials and methods

In the literature on cognitive differences between men and women, “sex” and “gender” have been used interchangeably. In recent years, a distinction between these terms has been clarified. “Sex” refers to biological and physiological characteristics such as hormones, chromosomes, and reproductive organs, whereas “gender” is a social construct, which includes norms, behaviors, and roles associated with being a man or a woman (WHO: https://www.who.int/health-topics/gender#tab=tab_1). We use the term “sex” in this paper.

### Social cognitive tests

*Emotion perception* was measured with Pictures of Facial Affect (PFA) and Emotion in Biological Motion (EmoBio); *social perception* with the Relationships Across Domains (RAD) test; and *ToM* with the Movie for the Assessment of Social Cognition (MASC) and Hinting Task. PFA (Frommann et al. 2003) consists of black-and-white photographs of Caucasian men and women expressing one of six basic emotions or neutral emotion. EmoBio (Heberlein et al. 2004; Vaskinn et al. 2016), a point-light measure, assesses the ability to identify and label emotions in moving bodies. RAD (Sergi et al. 2009) is a paper-and-pencil test where the test-taker answers questions about the relationship between a male and a female presented in short vignettes. The abbreviated Norwegian RAD (Vaskinn et al. 2017) was used. In the MASC test (Dziobek et al. 2006; Fretland et al. 2015), participants watch a movie of four characters during social interactions. The movie is repeatedly paused so that participants can answer questions about the thoughts, emotions, and intentions of a given character. The Hinting Task (Corcoran et al. 1995; Frøyhaug et al. 2019) presents ten vignettes describing a short encounter between two characters that ends with one of them dropping a hint. The test-taker is asked to identify this character’s intention.

### Other measures

Current intellectual level (*IQ*) was assessed with the two-test version of Wechsler Abbreviated Scale of Intelligence (WASI), consisting of the Matrix Reasoning and Vocabulary subtests (Wechsler 2007). We measured positive and negative symptoms with the Positive and Negative Syndrome Scale (PANSS: Kay et al. 1987).

### Participants

The study was conducted as part of the Thematically Organized Psychosis (TOP) research study at the Norwegian Centre for Mental Disorders Research in Norway. Data was collected at Oslo and Akershus University Hospital. Individuals with a SCID-verified (First et al. 1995) DMS-IV diagnosis of schizophrenia or schizoaffective disorders participated, along with healthy controls (HCs), recruited from statistical records. Participants provided informed consent to participate in the study, which was approved by the Regional Ethical Committee. Our participants with schizophrenia underwent assessments in a clinically stable phase.

In a cross-sectional case–control design, we analyzed four data sets used in previous studies. The core of the research protocol, also for cognitive tests, is largely the same across different studies subsumed under the TOP umbrella. However, different studies have used different social cognitive tests. Therefore, not everyone was assessed with all social cognitive tests. In particular, most HCs underwent assessments with just one or two of the five tests. Consequently, the number of participants depends on the social cognitive test in question. The exact numbers are provided in Table 1.

**Table 1:** Demographic and clinical information of included samples

	Sample 1 Vaskinn et al. 2018			Sample 2 Vaskinn et al. 2016			Sample 3 Frøyhaug et al. 2019		
	SZ (*n* = 91)	HC (*n* = 71)	Statistics	SZ (*n* = 84)	HC (*n* = 84)	Statistics	SZ (*n* = 30)	HC (*n* = 185)	Statistics
Age	29.1 (8.4)	29.3 (7.7)	*t* = 0.16 *p* = 0.872	29.0 (8.7)	30.8 (8.1)	*t* = 1.35 ns	33.5 (12.8)	34.7 (9.9)	*t* = − 0.61 *p* = 0.542
Sex (m/f)	57/34	42/29	χ^2^ = 0.20 *p* = 0.652	53/31	51/33	χ^2^ = 0.10 ns	18/12	104/81	χ^2^ = 0.15 *p* = 0.698
Education	12.2 (2.4)	14.2 (2.1)	*t* = − 5.34 *p* < 0.001	-	-	-	12.3 (2.1)	14.9 (2.1)	*t* = − 6.38 *p* < 0.001
WASI IQ	100.3 (13.2)	110.7 (10.8)	*t* = -5.59 *p* < 0.001	99.8 (13.6)	112.0 (11.5)	*t* = 6.32 *p* < 0.001	102.0 (13.9)	115.9 (9.3)	*t* = − 7.01 *p* < 0.001
PANSSpos	14.1 (4.8)	-	-	14.5 (4.6)	-	-	15.0 (5.2)	-	-
PANSSneg	14.7 (5.2)	-	-	14.6 (5.1)	-	-	17.0 (6.1)	-	-

*SZ* participants with schizophrenia, *HC* healthy control participants, *WASI IQ* Wechsler Abbreviated Scale of Intelligence, *PANSS* Positive and Negative Syndrome Scale

The samples in the Hinting Task/PFA analyses correspond to the Frøyhaug et al. (2019) samples, with the exception that two extra HCs have been added. The EmoBio analyses were undertaken on the study samples described in Vaskinn et al. (2016), whereas the samples for MASC analyses were used by Vaskinn et al (2018). Lastly, the schizophrenia sample for the RAD analyses was increased after the initial RAD publication (Vaskinn et al. 2017), but HCs are the same in the current study.

### Statistical analyses

Because the number of participants varied depending on the test, multivariate analyses were not possible. Five separate two-way analyses of variance (ANOVAs) examined the main effects of diagnostic group (schizophrenia vs. HC) and sex (males vs. females) and the group x sex interaction effect on social cognition (five social cognitive tests). Within-group sex differences were investigated with independent samples *t*-tests. For the latter we provide effect size estimates (Cohen’s *d*).

## Results

All five two-way ANOVAs identified significant main effects of diagnostic group, with HCs outperforming the schizophrenia group. Significant main effects of sex were present for the PFA test and Hinting Task. No interaction effects were statistically significant. The significant main effect of sex was reflected in a statistically significant sex difference among HCs on the independent samples *t*-test for PFA. Healthy males performed worse than healthy females, with a medium effect size (Cohen’s *d* = 0.44). This was the only independent samples *t*-test that yielded statistically significant results. Although not statistically significant, effect sizes for some within-group comparisons indicated medium-sized sex differences. For PFA, a medium-large sex difference (Cohen’s *d* = 0.74) was also present in the schizophrenia group. For the Hinting Task, a medium-sized, non-significant sex difference (Cohen’s *d* = 0.42) was present in the schizophrenia group. For RAD, a non-significant, medium-sized sex difference (Cohen’s *d* = 0.43) was seen among HCs. For all tests, males performed numerically worse than their female counterparts. See Table 2 for full information.

**Table 2:** Social cognition performance across sex in persons with schizophrenia (SZ) and in healthy control participants (HC)

	SZ			HC			Group x sex*
Emotion perception							
PFA (% correct)	Males (*n* = 18)	74.7 (9.6)	*t* = − 1.98 *p* = 0.058 Cohen’s *d* = 0.74	Males (*n* = 104)	81.5 (7.7)	*t* = − 2.97 *p* = 0.003 Cohen’s *d* = 0.44	G = 9.6, *p* = 0.002 S = 11.5, *p* < 0.001 GxS = 1.7, *p* = 0.196
	Females (*n* = 12)	81.9 (10.1)		Females (*n* = 81)	84.7 (6.8)		
EmoBio (range 0–1)	Males (*n* = 53)	0.79 (0.14)	*t* = − 0.47 *p* = 0.641 Cohen’s *d* = 0.11	Males (*n* = 51)	0.86 (0.08)	*t* = − 0.88 *p* = 0.378 Cohen’s *d* = 0.20	G = 16.0, *p* < 0.001 S = 0.7, *p* = 0.403 GxS = 0, *p* = 0.986
	Females (*n* = 31)	0.80 (0.16)		Females (*n* = 33)	0.88 (0.09)		
Social perception							
RAD (range 0–24)	Males (n = 55)	24.1 (5.9)	*t* = − 0.62 *p* = 0.540 Cohen’s *d* = 0.14	Males (*n* = 36)	28.2 (3.7)	*t* = − 1.54 *p* = 0.129 Cohen’s *d* = 0.43	G = 24.1, *p* < 0.001 S = 1.7, *p* = 0.199 GxS = 0.2,* p* = 0.699
	Females (*n* = 29)	24.9 (5.6)		Females (*n* = 20)	29.8 (3.4)		
Theory of mind (ToM)							
MASCtot (range 0–45)	Males (*n* = 57)	29.1 (7.3)	*t* = − 0.60 *p* = 0.553 Cohen’s *d* = 0.13	Males (*n* = 42)	34.7 (4.3)	*t* = -1.19 *p* = 0.238 Cohen’s *d* = 0.29	G = 36.1, *p* < 0.001 S = 1.2, *p* = 0.280 GxS = 0.02, *p* = 0.889
	Females (*n* = 34)	30.0 (6.2)		Females (*n* = 29)	35.8 (3.6)		
Hinting Task (range 0–20)	Males (*n* = 18)	14.5 (3.0)	*t* = − 1.14 *p* = 0.265 Cohen’s *d* = 0.42	Males (*n* = 104)	18.2 (1.8)	*t* = − 1.63 *p* = 0.105 Cohen’s *d* = 0.24	G = 59.0, *p* < 0.001 S = 5.5, p = 0.020 GxS = 1.8, *p* = 0.176
	Females (*n* = 12)	16.0 (4.2)		Females (*n* = 81)	18.6 (1.5)		

*SZ* participants with schizophrenia, *HC* healthy control participants, *PFA* Pictures of Facial Affect, *EmoBio* Emotion in Biological Motion, *RAD* Relationships Across Domains, abbreviated Norwegian version, *MASCtot* total score on the Movie for the Assessment of Social Cognition. *F-values with *p*-level reported for group (G), sex (S), and the group x sex (GxS) interaction

## Discussion and conclusions

In this study, we observed a consistent pattern of numerically higher social cognitive scores in women than in men. This was the case for all five tests and was evident in both HCs and participants with schizophrenia. A significant main effect of sex was however only found for two of the five social cognitive tests: PFA and the Hinting Task. For PFA, this was reflected in a statistically significant difference between healthy males and females and a non-significant difference between males and females with schizophrenia. For the Hinting Task, within-group sex differences were not statistically significant. The results replicate findings from large studies in the general population, concluding with better emotion recognition among women than men. For facial emotion perception, statistical significance, effect sizes, and inspection of the percentage correct responses all point to a female-advantage, across groups. The lack of statistically significant differences between men and women with schizophrenia aligns with the existing literature, which has provided limited evidence for such differences. Overall, the results largely confirmed our expectation of identifying few sex differences, with the exception of facial emotion perception.

The two significant main effects of sex were found in the same dataset (Frøyhaug et al. 2019). This was the study with the largest effect sizes among individuals with schizophrenia, i.e., for facial emotion perception and ToM, and had the smallest schizophrenia sample (*n* = 30). The individuals with schizophrenia in this sample do not appear to differ markedly from the schizophrenia samples in the other studies for demographic variables (see Table 1), but were perhaps slightly older. However, so were their healthy comparisons, thereby reducing the likelihood that age is responsible for the results. It should be noted though that an investigation of sex differences for the PFA test and Hinting Task among persons with schizophrenia should be repeated in a larger Norwegian sample.

In spite of few statistically significant sex differences, women did consistently outperform men. Larger samples and increased statistical power would likely render several of the within-group sex differences statistically significant. This especially pertains to social perception and ToM in healthy participants. Still, we are hesitant to interpret the consistently numerically higher scores in women as support of a *general* female-advantage in social cognition. There are several reasons for this. First, only one within-group sex difference was statistically significant (facial emotion perception among healthy participants), even without making adjustments for multiple comparisons. Second, most of the effect sizes (six of ten) were small or even negligible. The largest effect size was seen for facial emotion perception among participants with schizophrenia, where it was medium–large. Three effect sizes were medium (Cohen’s *d*s = 0.42–0.44). This was the case for facial emotion perception and social perception among HCs and for one ToM measure (Hinting Task) for participants with schizophrenia. The two sexes performed on the same level for the remaining tests. Third, the practical implications of the numerically higher scores in women may be limited, except for facial emotion perception, where our results indicate that men indeed have a disadvantage that matters. From this, one could hypothesize that sex differences in social cognition may be domain-specific, as is the case for nonsocial cognition (Zell et al. 2015; Hirnstein et al. 2023). If so, different performance levels between men and women would not be observed across social cognitive tests and functions. Based on the current findings, facial emotion perception emerges as the strongest candidate for a hypothesized sex-specific social cognitive function, should it exist.

Our reluctance to interpret the results as indicative of a general female-advantage in social cognition contrasts with conclusions reached in literature from the general population. In their meta-analysis of emotion recognition, Thompson and Voyer (2014) reported an overall effect size of *d* = 0.19 in favor of women, very similar to the effect size of *d* = 0.17 in Olderbak et al.’s study (2019). The sex difference for a nonsocial cognitive function, mental rotation, is substantially larger (*d* = 0.57) (Zell et al. 2015). Nevertheless, based on these social cognitive effect sizes, which are “small” according to common rules of thumb, it has been argued that females have a social cognitive advantage compared to males (Olderbak et al. 2019). In other words, smaller effect sizes than ours underlie claims of a female-advantage in social cognition. We concur with others in that it is important to remain cautious when interpreting sex differences (Rippon et al. 2021). Neuroscientific research has been criticized for overselling distinctions based on sex. The term *neurosexism* (Fine 2013) refers to scientific claims that there are fixed differences between the brains of men and women, claims which are used to explain or legitimize gender stereotypes. The limited statistical significance and few substantial effect sizes in the current study provide little evidence for a general female superiority in social cognition.

Our study has some limitations. Ideally, all tests should have been administered to the same sample. However, the current investigation consisted of secondary analyses of existing data taken from slightly different test protocols administered at different time points in the overall TOP study, so this was not possible. In addition, as mentioned, sample sizes were for the most part small. Larger samples likely would have increased the number of statistically significant sex differences.

Importantly, the sex difference we did find reflects a difference at the group level. Thus, not every individual will perform in line with the mean level of his or her group, i.e. sex in our context, and there will be an overlap in social cognitive performance between men and women. Therefore, tailoring social cognitive treatment based on sex is not warranted, and individualized treatment necessitates a social cognitive assessment.

In conclusion, this study found better facial emotion perception in healthy female participants and in women with schizophrenia, compared to their male counterparts. There were no sex differences for other social cognitive domains. Whereas sex-specific treatment may be recommended for some aspects of the schizophrenia illness (Fernando et al. 2020), the available evidence suggests that this is not the case for social cognitive impairment.
